# Physicochemical and Sensory Evaluation of Grain-Based Food

**DOI:** 10.3390/foods11091237

**Published:** 2022-04-26

**Authors:** Luca Serventi, Charles Brennan, Rana Mustafa

**Affiliations:** 1 Department of Wine, Food and Molecular Biosciences, Faculty of Agriculture and Life Sciences, Lincoln University, Lincoln 7647, New Zealand; 2 School of Science, RMIT University, P.O. Box 2474, Melbourne, VIC 3001, Australia; charles.brennan@rmit.edu.au; 3 Department of Plant Sciences, College of Agriculture and Bioresources, University of Saskatchewan, 51 Campus Drive, Saskatoon, SK S7N 5A8, Canada; rana.mustafa@gifs.ca

Grain-based food is a staple of the human diet. Whether it is cereals, legumes or pseudocereals, grain-based diets provide nutritional benefits. This can be in the form of macronutrients (starch, fibre, protein, and lipids) and micronutrients (minerals and vitamins), as well as bioactive peptides and phytochemicals [1]. Grains are used to develop bakery products, such as savory (bread, gluten-free bread, crackers, and pasta) and sweet (cakes and muffins) [2] in addition to plant-based beverages (milk alternatives), fermented products (such as yoghurt and fermented paste), extrudates and other snacks [3]. Furthermore, grain-based ingredients offer emulsifying, foaming and thickening abilities [4]. Raw materials include cereals (barley, corn, millet, rice, rye, spelt, wheat), legumes (beans, chickpeas, lentils, peas, and soybeans) and pseudocereals (amaranth, buckwheat, quinoa, and sorghum). The functionalities are numerous, spanning from health to taste. In order to fully exploit the nutritional potential of grain-based foods, consumer acceptance must be achieved. This will guarantee compliance. The acceptability of food can be studied both instrumentally and via sensory science.

The physicochemical evaluation of food can be performed via numerous techniques, exploring a broad range of functionalities. Foaming, emulsifying and thickening abilities can be assessed with specific tests. These results offer valuable information on grain-based ingredients capability to incorporate air (thus increasing volume), stabilize emulsion systems (air in water, oil in water, and water in oil) and increase viscosity, offering mouthfeel, while preventing syneresis and phase separation upon storage. Food products can be assessed for texture, rheology (viscosity and pasting properties), thermal properties (through differential scanning calorimetry and thermogravimetric analysis) as well as water mobility (nuclear magnetic resonance) in addition to image analysis (microscopy and particle size) [5,6,7].

Sensory evaluation includes both consumer preference and trained panels. Consumer panels reveal human preferences for appearance, aroma, taste and texture. It can be performed traditionally in sensory booths, or with modern techniques such as immersive technologies and augmented reality. The goal is to predict consumers’ acceptability of food products. Tests include hedonic scale, threshold, the triangle test and others [8]. Trained panels are used in focus groups, which allow us to study specific attributes with experts of each type of food. This technique is useful in describing new foods as well as in the investigation of their shelf-life stability [9].

Sustainable food supply is a contemporary issue of high relevance. Societies must be able to produce food sustainably, meaning with lower environmental impact (less carbon and water footprint, and minimized land use), high nutritional quality, safety and sensory quality. Local crops, plant-based foods and upcycling of processing side streams are three answers to this call. The application of grains to non-traditional foods (egg, dairy, meat alternatives) and traditional (bakery) offers new ways to deliver nutrition along with high taste. Examples of upcycling include *aquafaba* and *liluva* (the processing water of legumes) used in egg replacers or as alternatives to hydrocolloids [10,11,12]. 

In recent years, there has also been attention focused on the bioactive ingredients of cereal grains and their benefits in terms of nutritional well-being [13,14]. However, these bioactive ingredients (fibre and phenolic compounds for instance) can affect the physical nature of foods as well as their sensory quality [15]. This is particularly the case when considering the use of wholegrains in foods [16].

Therefore, the aim of this Special Issue is to illustrate the latest scientific advances in the field of grain-based foods, investigating their physicochemical properties and sensory qualities. The focus is on sustainable solutions such as local crops (amaranth, ancient grains, buckwheat, maize, quinoa, rice, and spelt), plant-based products (yoghurt and egg alternatives) and upcycled ingredients (*aquafaba*, *liluva*, and pomace).
